# Kinesiologist-guided functional exercise in addition to intradialytic cycling program in end-stage kidney disease patients: a randomised controlled trial

**DOI:** 10.1038/s41598-020-62709-1

**Published:** 2020-03-31

**Authors:** Špela Bogataj, Jernej Pajek, Jadranka Buturović Ponikvar, Vedran Hadžić, Maja Pajek

**Affiliations:** 1 University Medical Centre, Department of Nephrology, Ljubljana, 1000 Slovenia; 2 University of Ljubljana, Faculty of Sport, Ljubljana, 1000 Slovenia; 3 University of Ljubljana, Faculty of Medicine, Ljubljana, 1000 Slovenia

**Keywords:** Diseases, Health care, Nephrology

## Abstract

Intradialytic cycling is a widely used workout mode, whereas added benefit of other exercise modalities remains unknown. This is the first randomised controlled trial on the effects and sustainability of functional training and counselling in addition to intradialytic cycling. Patients were randomly assigned to a kinesiologist-guided functional training in addition to intradialytic cycling (n = 20, experimental group) or intradialytic cycling only (n = 20, control group) over 16 weeks. The experimental group attended predialysis functional exercise in the first eight weeks and afterward performed functional training at home for the next eight weeks. The primary study endpoint was 10-repetition-sit-to-stand test time at eight weeks: at this test, the experimental group improved significantly better than controls (−4.5 ± 1.9 s, 95%CI −8.4 to −0.7; P = 0.021), which was maintained at week 16 (−4.7 ± 2.1 s, 95%CI −9.0 to −0.3; P = 0.037). At week 8, the experimental group significantly outperformed controls also at handgrip strength (P = 0.004), lower body flexibility test (P < 0.001), balance test (P < 0.001), and upper body flexibility test (P = 0.003). At week 16, superior results of the experimental group in secondary end-points remained preserved for handgrip strength, balance, and upper body flexibility tests. Functional training with exercise counselling meaningfully improves physical performance and successfully prepares patients for sustainable home exercise.

## Introduction

Intradialytic exercise on a customized ergometer (cycling) is currently the most common mode of in-centre exercise among haemodialysis (HD) patients^1,2^. This exercise mode is well feasible, time-efficient, can be easily supervised^1^ and has shown numerous improvements in aerobic capacity^3–6^, functional performance^7–9^, HD efficiency (improved Kt/V)^1^, and quality of life^1,5,7^. It could be perceived as the current standard of physical training in the HD population. However, the comparative data on the efficiency and sustainability of various types of exercise modes are lacking. With the previous studies showing a minimal efficacy of intradialytic cycling on the functional performance of HD patients^7,9–12^, we need innovative strategies to improve the outcome and sustainability of dialysis exercise programs.

Functional training simulates activities of daily living^13^ and targets the neuromuscular system to train movements that activate both the nervous system and the muscle groups^14^. Functional training is performed as a combination of lower and upper body movements including various multi-joint activities^15^. A systematic review among older adults, including 13 trials with 1139 participants, demonstrated positive effects of functional training on muscle strength, physical functioning, and activities of daily living^16^. The authors stated that functional training, which imitates specific performance, confers the best performance gains. HD patients have largest functional deficits in flexibility, balance, and lower extremity functions^17^. These are all vital motor abilities for supporting the activities of daily living, and their loss can lead to the patient’s dependence on the carer. Successful strategies that could prevent this, such as functional training implementation, would meet a significant need of dialysis patients.

Effectiveness of functional training in end-stage kidney disease patients on dialysis has not been investigated so far. Furthermore, the level to which dialysis patients can be prepared to continue with exercise routines in their home environment and the sustainability of exercising in an unsupervised home environment is mostly unknown in this population. Therefore, our aim was to determine the effects of supervised functional training and counselling added to the basic program of intradialytic cycling on physical performance in HD patients. We intentionally chose to supplement intra-dialytic cycling with pre-dialysis functional training to verify this strategy as a possible improvement in content and volume of contemporary well-established intradialytic cycling programs. An additional argument for the retention of cycling in the experimental group is its putative effects in the prevention of HD related myocardial stunning^18^. In the extension phase of this trial, we sought to determine the achievable level of transfer of mastered functional exercise routines to a home environment on non-dialysis days in order to evaluate the sustainability of this approach. We hypothesized that pre-dialysis functional training and exercise counselling in addition to intra-dialytic cycling as compared to intra-dialytic cycling only would (i) significantly improve physical performance at tests that reflect the activities of daily living, and (ii) that this improvement in physical performance would be sustained after a period of functional training in an unsupervised home environment.

## Methods

This was a prospective, randomised, controlled, interventional trial comparing two strategies of exercise prescription and counselling in prevalent HD patients. Seventy-three HD patients were approached in the HD units of the University Medical Centre in Ljubljana, Slovenia. The inclusion criteria were the following: end-stage kidney disease, renal replacement therapy with HD > 3 months, age 18–90 years, capable of independent walking and feeding, in a stable medical condition. Criteria for non-inclusion were: chronic malignant or infectious disease, uncontrolled arterial hypertension with an average of the last five in-centre pre-dialysis blood pressure values above 180/100 mm Hg, angina pectoris of Canadian Cardiovascular Society grade 2–4, New York Heart Association heart failure grade 3 or 4, the presence of a psychotic illness or a mental disability, a history of limb amputation (more than 2 fingers on the lower limb and/or more than 2 fingers on the upper limb) or any other condition that might cause clinical instability of the patient (e.g. repetitive gastrointestinal haemorrhages, liver cirrhosis with frequent exacerbations, advanced dementia with poor cooperation of the patient). Study exclusion (withdrawal) criteria contained any intercurrent illness or trauma that prevented the patient to continue with the exercise program for a period longer than 14 days, the occurrence of an acute illness lasting more than 3 weeks or ending less than 3 weeks before the end of the study, or if the patient has had prescribed medication for it at the end of the study, diagnosis of malignant disease during the course of the research and withdrawal of the consent to participate. National Medical Ethics Committee approval (Ministry of Health, Republic of Slovenia, approval document number 0120-97/2017-3 KME 68/03/17) and written informed consent were obtained in all cases. The study complies with the Declaration of Helsinki, as revised in 2013. The study was registered at ClinicalTrials.Gov (Clinicaltrials.gov identifier: NCT03334123) on November 7, 2017.

The primary endpoint was the 10 repetition sit-to-stand test time (STS-10) at week 8, which measures lower limb strength^19^. The rationale for the choice of this endpoint was based on our previous research showing the superiority of this test in sensitivity to uraemia effects, the strength of association with activities of daily living, and a relatively large deficit at this test in dialysis patients compared to other physical performance tests^17,20^. The secondary endpoints were: the aerobic capacity assessed by the six-minute walk test^21^, hand-grip strength^22^ assessed with calibrated hydraulic hand dynamometer (Jamar, Patterson Medical, Warrenville, Ilinois), lower body flexibility by sit-and-reach test^23^, balance by Stork test^20^ on a foam pad (Airex, Sins, Switzerland) and upper body flexibility with back scratch test^24^. All tests were executed as previously described^17,25,26^ in a fixed order to minimize patient fatigue. Before the tests, we measured height, weight, and body composition using bioimpedance analysis (Body Composition Monitor, Fresenius AG, Bad Homburg, Germany). The first functional test executed was a six-minute walk test followed by a hand-grip strength test, sit-and-reach test, balance Stork test, and back scratch test. It took approximately 40 minutes for a patient to complete all outcome assessments (including rest periods). The testing was performed on dialysis-free days: patients who were on dialysis on Tuesday-Thursday-Saturday schedule were tested on Friday and patients who were dialyzed on Monday-Wednesday-Friday schedule were tested on Saturday. Outcomes were assessed at three time points: before the intervention (T1), after eight weeks (T2, end of phase 1), and after 16 weeks (T3, end of phase 2). The same assessors were assigned to individual endpoint assessments at all times and were blinded to treatment allocation. Patients and in-center dialysis care providers were not blinded.

After baseline testing, the patients were randomised using a computer program and allocated in a 1:1 ratio to the experimental group (EXP) or the active control group (CON). We concealed allocation to patients and dialysis staff before the start of the study by including and allocating patients according to a list, which was assessed by researchers only. For the first eight weeks of intervention (phase 1), the experimental group performed guided functional training led by a kinesiologist prior to each HD procedure. During these sessions, they received counselling to accurately master the functional exercise routines and transfer these skills to a home environment in the second phase of the study. Besides, they performed a cycling program during dialysis on the customized ergometer (Model B’fit Mini, Lemco, Denmark) during the first half of the dialysis procedure. The control group performed the intradialytic program of cycling only, with the same instructions as the experimental group. Specifically, intradialytic cycling was supervised by the same kinesiologist who aimed to continuously progress the cycling load (resistance) or time with maintaining the rate of perceived exertion of 4th to 5th grade on a 10-grade Borg scale. Initial intradialytic cycling duration was set to 15 minutes with a gradual increase in time and intensity to reach the duration of up to 60 minutes. The speed of increase in load and duration was individualised according to each patient rated perceived exertion (RPE) response and their motivation. This prescription strategy was the same in both study groups. RPE was graded using the Borg 10-grade scale, which is useful for the evaluation of exercise intensity in all patients regardless of the presence of arrhythmias, chronotropic insufficiency, and demographic characteristics^27^.

The pre-dialysis functional training lasted for up to 30 minutes before each HD session. The number of repetitions, sets, and load were adjusted to each individual in order to achieve the desired intensity of RPE of 7^th^ to 8^th^ grade on a modified Borg scale (range 0 to 10). Pre-dialysis functional training, exercise counselling, and intradialytic cycling were prescribed and monitored by a kinesiologist. Functional training consisted of different full-body exercises that train flexibility, strength, balance, coordination, power, and endurance. They cover the three fundamental movement planes (frontal, sagittal, rotational) and include the most important movement patterns (pull, push, lift, squat, lunge)^28^. First, we started with approximately five different exercises with ten repetitions of each in two sets without the extra load. Second, we gradually either increased the number of repetitions or added load. We aimed to achieve the completion of three sets of each exercise with 10–15 repetitions. However, exercise progression was always individualised. Functional training included a full range of motion exercises with additional weights tailored to the individual’s capacity. In the warm-up, patients performed light cardiovascular exercises and exercises for coordination and balance. The main part of the functional training consisted of varieties of lunges, squats, push-ups, pulls, pushes, and lifts adapted to each individual’s abilities. The cool-down period included light cardiovascular exercises combined with stretching. Exact functional training content is given in Supplementary Table S2.

**Figure 1:**
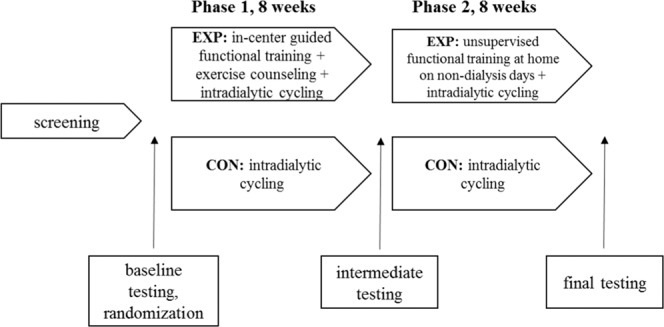
Flow diagram of the randomized controlled study design.

In the second study phase, pre-dialysis functional training was discontinued, and the experimental group patients were advised, monitored, and motivated to perform the functional exercise at home on non-dialysis days three times a week. On dialysis days, we assessed compliance and discussed the issues of home functional exercise giving feedback, advice, and motivation. In the first study phase, exercise counselling was given at the time of functional training. The patients received instructions on how to correctly perform an exercise, how to modify an exercise, and how to adjust the resistance/load. In the second study phase, where they performed the functional exercises at home, they received counselling and motivation at each dialysis session. They received a written individualized exercise program describing and illustrating the exercises and discussed with them how to implement them in their home environment. In cases where they did not have any exercise equipment, we suggested using alternatives like water bottles, towels, couch, chair, and table. At every dialysis session of study period 2 they reported the details about the exercise performed. We focused on motivating the patients to stay engaged in the exercise process by discussing the barriers to exercise, setting goals, monitoring safety, and identifying and solving intercurrent problems. Both groups continued the program of intra-dialytic cycling throughout the second phase of the study (see Fig. 1 for the study flow diagram).

A previous study in the Slovenian dialysis patient sample^17^ showed the average result in the 10 repetition sit-to-stand test of 19.3 s with a standard deviation of 7.1 s. The study demonstrated that the adjusted calculated difference between healthy controls and dialysis patients was 29%. According to this, we estimated a clinically meaningful detectable improvement to 30% (5.8 s). With an alpha error of 0.05 and a beta error of 0.2, a total of 50 subjects were required for the analysis of the variance between the two equally large groups. Further, with the expected 10% dropout, we calculated an overall sample size of 56 patients at randomisation.

**Figure 2:**
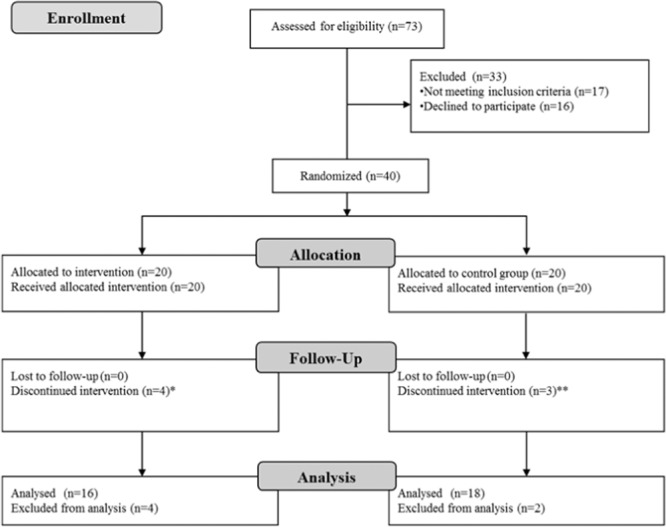
CONSORT (Consolidated Standards of Reporting Trials) diagram. *death (n = 1), sepsis (n = 1), fall and osteomuscular injury (n = 1), discontinued on patient’s demand; exacerbated symptoms of spinal stenosis (n = 1), **discontinued on patient’s demand (n = 1), coronary artery disease exacerbation with exertional dyspnea (n = 1), transplantation (n = 1).

Analysis of covariance (ANCOVA) was used to test for differences between the groups with the baseline value as a covariate. We used paired t-test to compare changes over time in within-group analyses. In the case of non-symmetric data distribution, we transformed the data using natural log transformation. All tests were 2-sided, carried out using SPSS, version 22 (SPSS Inc., Chicago, IL, USA), and assessed at the P < 0.05 level of significance. We tried to obtain endpoint results from all patients regardless of the compliance with the study intervention, changes of study arms or maintenance of participation throughout the trial intervention period. Subjects who were withdrawn from the study were not included in the analysis since their withdrawal causes and events either disabled the patients to perform endpoint assessment measurements or they refused to participate (see Fig. 2).

## Results

### Patient flow through study phases and exercise adherence

From November 2017 to February 2019, 40 patients were randomised, and 34 completed the study (Fig. 2). Table 1 summarizes patient baseline characteristics.

**Table 1:** Demographic and clinical characteristics.

	All participants (n = 40)	Experimental group (n = 20)	Control group (n = 20)
Age (years)	63.6 ± 12.5	65.2 ± 12.1	61.9 ± 13.0
Male sex (%)	55%	60%	50%
Height (cm)	167.9 ± 9.8	168.4 ± 9.6	167.5 ± 10.2
Weight (kg)	72.1 ± 15.8	72.6 ± 16.1	71.7 ± 15.9
Dialysis vintage (years)	7.4 ± 7.7	7.4 ± 8.1	7.5 ± 7.3
Weekly dialysis duration (h)	12.9 ± 2.3	12.5 ± 2.7	13.3 ± 1.9
Type of treatment (HD vs. HDF)	15 vs. 25	9 vs. 11	6 vs. 14
Lean tissue index (kg/m^2^)	13.3 ± 2.6	13.6 ± 3.2	12.9 ± 2.0
Fat tissue index (kg/m^2^)	11.5 ± 5.4	11.4 ± 4.8	11.6 ± 6.1
Phase angle (°)	5.0 ± 0.9	5.2 ± 0.9	4.7 ± 0.9
BIA assessed overhydration (L)	1.4 ± 1.9	0.9 ± 1.1	1.9 ± 2.4
Hemoglobin (g/L)	120.2 ± 9.6	118 ± 7.3	122 ± 11.3
Albumin (g/L)	39.5 ± 2.9	39.4 ± 3.2	39.6 ± 2.5
C-reactive protein (mg/L)	9.2 ± 17.2	7.4 ± 14.2	11.0 ± 20.0
Phosphorous (mmol/L)	1.5 ± 0.5	1.5 ± 0.5	1.5 ± 0.5
Systolic blood pressure (mm Hg)	143 ± 15.4	141 ± 16.1	144 ± 14.93
Diastolic blood pressure (mm Hg)	81 ± 10.1	78 ± 10.2	84 ± 9.38
Serum pre-dialysis creatinine (qmol/L)	751 ± 148	750 ± 156	751 ± 143
Urea (mmol/L)	24 ± 5.8	22.6 ± 5.7	25.4 ± 5.73
Davies comorbidity grade 0/1/2 (*n* (%))	27 (67.5)/9 (22.5)/4 (10)	13 (65)/6 (30)/1 (5)	14 (70)/3 (15)/3 (15)

Note: Values are expressed as mean ± SD, number of subjects (percent). There were no statistically significant differences between the groups. Blood pressure was defined as the mean of the last three pre-dialysis blood pressure values. Phase angle measurements were performed with an 800 μA current at a frequency of 50 kHz.

Abbreviations: n, number of subjects; BIA, bioimpedance performed using Body Composition Monitor, Fresenius AG, Bad Homburg, Germany.

We defined adherence to training programs as the total number of completed exercise sessions over a total number of sessions offered/advised (see Table 2). Overall adherence was high, reaching more than two-thirds of prescribed exercise volumes. There were no significant between-group differences in cycling adherence, but we noticed a significantly (P = 0.034) lower adherence to home-based functional training routines compared to in-centre functional training in the experimental group.

**Table 2:** Adherence to training programs.

	Phase 1		Phase 2	
	Experimental group (n = 16)	Control group (n = 18)	Experimental group (n = 16)	Control group (n = 18)
Intradialytic cycling	90% ± 12%	87% ± 10%	82% ± 19%	82% ± 13%
Functional training	87% ± 12%	n/a	73% ± 21%^*^	n/a
Cycling time (min/session)	30.5 ± 8.3	31.8 ± 7.8	46.6 ± 17.0^**^	44.4 ± 12.8^**^

Note: Values are expressed as mean ± SD and characterize adherence to training programs defined as the total number of completed exercise sessions in contrast to a total number of sessions offered/advised. Abbreviations: n, number of subjects; n/a, not applicable; *P < 0.05 indicates significant within-group difference; **P < 0.01 indicates significant within-group difference.

### Primary outcome: changes in 10-repetition-sit-to-stand performance

**Figure 3:**
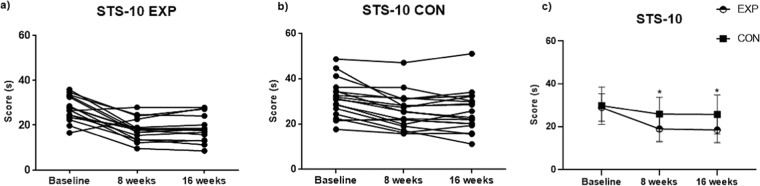
Results of 10 repetition sit-to-stand test over time Note: (**a**) individual changes in 10 repetition sit-to-stand test for experimental group; (**b**) individual changes in 10 repetition sit-to-stand test for the control group; (**c**) changes of the mean in 10 repetition sit-to-stand test for both groups, summarized; *P < 0.05 indicates significant between-group difference. Abbreviations: EXP, experimental group; CON, control group; STS-10, 10 repetition sit-to-stand test.

At week 8 and week 16, the time of the 10-repetition-sit-to-stand test was significantly shortened in both groups (Table 3, Fig. 3). Baseline adjusted ANCOVA analyses (Table 4) revealed a significant between-group mean difference of −4.5 ± 1.9 s (95% CI −8.4 to −0.7 s; P = 0.021) at week 8 and −4.7 ± 2.1 s (95% CI −9.0 to −0.3 s; P = 0.037) at week 16 in favor of the experimental group. Within-group baseline adjusted the relative change in 10 repetition sit-to-stand test time for the experimental group at week 8 was −30% and −15% for the control group (P = 0.04).

**Table 3:** Physical performance results during the study periods.

Variable	Group	Baseline	8 weeks	16 weeks
10 repetition sit-to-stand test (s)	EXP	28.9 ± 6.5	18.9 ± 5.9^**^	18.5 ± 5.8^**^
	CON	29.8 ± 8.8	25.9 ± 7.9^**^	25.7 ± 9.1^**^
6-minute walk test (m)	EXP	481 ± 99.6	551 ± 90.8^**^	579 ± 96.7^**^
	CON	482 ± 96.8	498 ± 87.4^**^	511 ± 100.2^*^
Hand-grip strength test (kg)	EXP	28.6 ± 8.1	34.4 ± 9.1^**^	33.4 ± 9.5^*^
	CON	28.3 ± 6.1	27.7 ± 5.2	26.2 ± 5.7
Sit-and-reach test (cm)	EXP	9.5 ± 7.1	14.3 ± 8.8^**^	12.0 ± 9.1^*^
	CON	3.8 ± 10.4	4.4 ± 9.6	4.6 ± 10.5
Stork test (s)	EXP	4.3 ± 9.4	9.1 ± 11.0^**^	10.5 ± 14.1^*^
	CON	7.1 ± 9.2	9.0 ± 13.5	9.3 ± 13.9
Back scratch test (cm)	EXP	−14.0 ± 12.4	−8.0 ± 12.2^**^	−9.0 ± 12.7^**^
	CON	−10.9 ± 16.4	−10.0 ± 17.5	−8.9 ± 18.2^*^

Note: Values are expressed as mean ± standard deviation. There were no statistically significant differences between groups at baseline. Abbreviations: EXP, experimental group; CON, control group. *P < 0.05 indicates significant within-group difference compared to the baseline value. **P < 0.01 indicates significant within-group difference compared to baseline value.

**Table 4:** Results of 8-week and 16-week ANCOVA: the difference between the experimental and control group.

Variable	At 8 weeks		At 16 weeks	
	Difference (95% CI)	P value (EXP-CON)	Difference (95% CI)	P value (EXP-CON)
10 repetition sit-to-stand test	−4.5 ± 1.9 (−8.4 to −0.7)	0.021	−4.7 ± 2.1 (−9.0 to − 0.3)	0.037
6-minute walk test	9.5 ± 14.4 (−19.7 to 38.7)	0.514	31.8 ± 19.7 (−8.4 to 72.0)	0.117
Hand-grip strength test	3.7 ± 1.2 (1.3 to 6.2)	0.004	4.3 ± 1.6 (1.1 to 7.5)	0.01
Sit-and-reach test	5.8 ± 1.4 (2.9 to 8.6)	<0.001	2.7 ± 1.4 (−0.1 to 5.5)	0.054
Stork test (ln value)	0.7 ± 0.2 (0.4 to 1.1)	<0.001	0.5 ± 0.2 (0.2 to 0.9)	0.005
Back scratch test	5.8 ± 1.8 (2.2 to 9.5)	0.003	3.8 ± 1.7 (0.4 to 7.3)	0.032

Note: Values are expressed as mean ± standard deviation. Analysis for Stork test was performed on natural logarithm transformed values. All significant between-group differences with ANCOVA adjusted test were also significant with unadjusted between-group ANOVA test (P < 0.05). Abbreviations: EXP, experimental group; CON, control group; ANCOVA, analysis of covariance.

### Secondary outcomes

Within-group changes in secondary outcomes are presented in Table 3 and between-group differences in Table 4. There were significant improvements in the 6-minute walk test distance in experimental as well as in the control group at the end of both study phases with no significant differences between groups. All remaining tests were significantly improved in the experimental group while there were no significant changes in the control group (except for the back scratch test, where there was a significant improvement in the control group at the end of phase 2). Between-group comparisons have shown a significant benefit of the experimental intervention over the control group in upper extremity strength (hand-grip strength test), balance (Stork test), upper and lower body flexibility (sit-and-reach test and back scratch test). In all these domains, the significant benefit gained in the first study phase remained preserved in the second study phase, except for the borderline P value of the seat-and-reach test (see Tables 3 and 4 for exact numerical data and significances). No significant changes were observed for blood pressure and body composition parameters (Supplementary Table S1).

### Adverse events and specific observations

Observed adverse events are presented in Table 5. They were mostly composed of isolated hypotension, fatigue, and joint/low back pain episodes. There was a single death event due to severe sepsis in a long-term diabetic patient. No major cardiac events were observed.

**Table 5:** Adverse events encountered during the time of the study.

Adverse event	EXP (n)	CON (n)	Comments
Low back pain	2	1	one early termination of cycling session and one of functional training due to lower back pain; one patient discontinued exercise due to worsening of spinal stenosis
Vascular access hematoma	1	1	two patients could not perform the cycling session due to hematoma not related to exercise
Hypotension episodes	0	2	one patient presented hypotension after a cycling session and one during the 6-min walk test
Fatigue episodes	3	2	three patients from the EXP group and two from the CON group prematurely stopped the exercise session due to fatigue
Muscle/joint pain	0	1	one patient’s intensity had to be reduced due to the exacerbation of hip pain
Hospitalization	2	1	two patients missed a few exercise sessions due to hospitalization not related to exercise; one patient discontinued intervention due to sepsis occurrence
MACE	0	0	
Death	1	0	one death (sepsis)

Abbreviations: EXP, experimental group; CON, control group; n, number of events; MACE, major adverse cardiac events.

Several important practical and logistic observations came up to our attention during the study execution period. The main reasons for skipping the cycling sessions were fatigue, high blood pressure, hypotension, presence of vascular access-related haematoma, and dizziness. Similar problems were reported for pre-dialysis functional training sessions, with the added limitation of late transportation to the dialysis unit, consequently not being able to attend the pre-dialysis functional exercise. Patients sometimes felt knee pain during or after cycling due to inappropriate body position while exercising. Some of them were afraid to increase paddling speed because of the fear of causing a hematoma with an inadvertent needle trauma. Some patients expressed concerns about sweating during cycling and wanted to avoid lying in sweaty clothes until the end of their dialysis. As for the home functional exercise program, patients reported limitations in terms of a lack of indoor space, motivation for exercise and not knowing if exercises were performed correctly.

## Discussion

In this study, we examined the effect of functional training added to basic intradialytic cycling and investigated the feasibility and sustainability of functional exercise transfer to an unsupervised home environment. Results demonstrate significant superiority of experimental training strategy concerning the primary study endpoint associated with lower extremity performance. Superiority in balance, hand-grip strength and upper body flexibility was also shown. Although significant intra-group improvements in 6-minute walk test distance were found in the experimental group, the effects did not reach statistical significance compared to the control group. Importantly, patients were able to maintain gained positive effects of supervised in-centre functional training in the second phase of the study, where they successfully transferred mastered functional training routines to their home on non-dialysis days.

In the experimental group, we found a large 10 second (36%) absolute mean improvement in the 10 repetition sit-to-stand test time (Table 3), which was larger than the results found in previous studies with improvements ranged from 2.5 to approximately 6 seconds^29–32^. We believe that this substantial effect was due to the relatively modest baseline physical performance, which is typical for dialysis subjects, relatively large training input in terms of volume and intensity, and due to individualisation of exercise program to each individual. The magnitude of this change is not only clinically meaningful since it covers more than the relative magnitude of difference to untrained healthy subjects, but it is also significantly and meaningfully larger than the improvement found in the control intra-dialytic cycling group. Similarly, more than 4 kg improvement in hand-grip strength represents significant clinical value having in mind that hand-grip strength is a reliable indicator and prognostic marker in the assessment of overall strength and outcome in kidney and geriatric patients^33,34^. As evident from Table 3, flexibility parameters were also significantly improved compared to controls. This improved flexibility can be beneficial for musculoskeletal pain management^35^. Prolonged time in the Stork balance test may be helpful for the prevention of falls and fractures^36^.

Sustainability of exercise at home is a highly relevant issue for many dialysis programs since the availability of kinesiologists or physical therapists for indefinite in-centre guidance may be limited. At the same time, HD patients usually demonstrate lower levels of physical functioning and exercise capacity^37^, and thus increasing their level of activity on non-dialysis days is generally needed. Our results confirmed the effectiveness and sustainability of functional training transfer to a home environment since a performance at all tested functional domains at the end of the second home-based phase remained significantly improved over baseline results. Self-reported adherence to home functional exercise at 73% was satisfactory and in accordance with preserved benefit noted at final testing. To the best of our knowledge, we could only find one other trial in HD patients that looked into the success of the transfer of mastered exercise routines from the supervised medical to the home environment^38^. The study investigated the effects of exercise training and counselling comparing it to standard care. They were able to show success in maintaining gained improvements during the home exercise phase, however in a much younger, less generally representative sample. Exercise counselling without the addition of supervised exercise did not demonstrate significant effects in another study^39^.

Regarding home-based exercise efficacy, other studies found similar positive findings in cardiac patients^40^ and patients with chronic obstructive pulmonary disease^41^. Further trials would need to explore the sustainability of this strategy on a longer-term, since our results may not be extrapolated to significantly longer periods of home training. A relatively short time of intervention is also one of the probable reasons for no impact of the intervention on the body composition indices. Additionally, a possible impact on the reduction of morbidity and mortality needs to be assessed.

Our findings need to be interpreted in light of a few limitations. Due to several issues (slower than anticipated recruitment, unsuspected logistic problems with interrupted transport service of patients - transport services not willing to adjust the time of transport for training needs, and space constraints with loss of exercise rooms due to unplanned renovation needs), we reached 71% of the planned sample size. According to pre-study power calculation presumptions, this sample size gives a beta error estimate of 0.34; however, our final results have shown that this was a too conservative estimation with the final post-hoc beta error result of 0.21. In light of a clearly significant positive result of the trial, these post-hoc beta error considerations may, however, be judged as less relevant. Additionally, we were not able to entirely prevent contacts and a possible exchange of experience between participants. This way some dissemination of functional training routines may have happened from experimental to control group participants, although during the interviews and follow-ups, no such activity was reported from the control group patients. We tried to avoid detection bias by blinding the outcome assessors to group allocation. We could not blind the participants because they were aware of which exercise they performed. The same goes for care providers as they could see which patient is performing which exercise program.

On the other hand, we were able to avoid some pitfalls of previous studies such as nonrandomised designs^30,42,43^, not reporting whether outcome assessors were blinded, failure to report exercise adherence^44,45^, and not using the intention-to-treat analysis^31,46,47^. We employed a structured, individualised, perceived effort-controlled exercise intervention, which took into consideration baseline performance level and thus were able to follow through even frail elderly patients. While many of exercise sessions in previous studies were nurse-led^38,48^, the special skills of a kinesiologist employed in our study may be a contributory factor in creating an improved exercise culture among patients since kinesiologist has specific knowledge and skills of prescribing, progressing and monitoring exercise programs. Associated with this, although several systematic reviews^1,49–55^ reported the attrition level for exercise interventions in CKD to be around 30–40% and the recruitment rate around 40% our recruitment success was larger (55%) and attrition level lower (16%). We believe that devoted personal contact of the kinesiologist and nurses combined with strong support from in-centre attending physicians was the key to this improved recruitment and attrition rates. Additionally, spouses and close family members were informed about study purpose and invited to support the patient in some cases.

The majority of previous systematic reviews and meta-analysis have examined the effects of exercise in general within the HD population^49,51,53,55,56^. Moreover, the most recent meta-analysis showed the most beneficial effects of combined (strength and aerobic) exercise in HD patients^57^. This study revealed novel findings regarding the exercise modality used in HD patients. Our results showed that functional training on top of intradialytic cycling resulted in significant improvement in physical performance. Compared to intradialytic cycling alone, it resulted in significant and clinically meaningful improvements in lower extremity strength, balance, hand-grip strength, and flexibility. Most importantly, under the guidance of a kinesiologist, patients successfully transferred mastered functional training routines to an unsupervised home environment. With continued support through motivational monitoring, they were able to maintain a satisfactory adherence to home exercise and preserved gained benefits throughout the additional two months of unsupervised exercise. Significant treatment effects found in our study set the stage for further research that should focus on longer follow-up and a larger sample, also including clinical events and patient-reported outcomes. Currently, we will use hereby-gathered evidence to argument institution and funding of the expert teams providing functional exercise interventions to meet this important need of the Slovenian dialysis population.

## Supplementary information

Supplementary Information.

## Data Availability

The data that support the findings of the presented study is available from the University Medical Centre, Ljubljana, Slovenia. Data are available upon request.
