# Author Correction: Kinesiologist-guided functional exercise in addition to intradialytic cycling program in end-stage kidney disease patients: a randomised controlled trial

**DOI:** 10.1038/s41598-020-67057-8

**Published:** 2020-06-23

**Authors:** Špela Bogataj, Jernej Pajek, Jadranka Buturović Ponikvar, Vedran Hadžić, Maja Pajek

**Affiliations:** 1 University Medical Centre, Department of Nephrology, Ljubljana, 1000 Slovenia; 2 University of Ljubljana, Faculty of Sport, Ljubljana, 1000 Slovenia; 3 University of Ljubljana, Faculty of Medicine, Ljubljana, 1000 Slovenia

Correction to: *Scientific Reports* 10.1038/s41598-020-62709-1, published online 31 March 2020

The original version of this Article contained errors.

An affiliation was omitted for Jernej Pajek. The correct affiliations for Jernej Pajek are listed below:

University Medical Centre, Department of Nephrology, Ljubljana, 1000, Slovenia

University of Ljubljana, Faculty of Medicine, Ljubljana, 1000, Slovenia

In addition, Table 3 contained errors where the data given for the Back scratch test (cm) at 8 weeks was incorrect owing to the inadvertent omission of minus symbols:

8.0  ±  12.2** and 10.0  ±  17.5

now reads:

−8.0  ±  12.2** and −10.0  ±  17.5

Finally, the author names in Reference 57 were incorrectly given as:

Bogataj, P., Pajek, B. P. & Paravlic Exercise-Based Interventions in Hemodialysis Patients: A Systematic Review with a Meta-Analysis of Randomized Controlled Trials. *J. Clin. Med*. **9**, 43 (2020).

This has been corrected to read:

Bogataj, Š., Pajek, M., Pajek, J., Buturović Ponikvar, J. & Paravlic, A. Exercise-Based Interventions in Hemodialysis Patients: A Systematic Review with a Meta-Analysis of Randomized Controlled Trials. *J. Clin. Med*. **9**, 43 (2020).

These errors have now been corrected in the HTML and PDF versions of this Article, and in the accompanying Supplemental Material.

