# Supplementary material for: Kinesiologist-guided functional exercise in addition to intradialytic cycling program in end-stage kidney disease patients: a randomised controlled trial
Source: Sci Rep. 2020 Mar 31;10:5717. doi: 10.1038/s41598-020-62709-1 (PMC7109131; doi:10.1038/s41598-020-62709-1)
Supplement: Supplementary file 1 — Supplementary Information. [file 41598_2020_62709_MOESM1_ESM.docx]

**Kinesiologist-guided functional exercise in addition to intradialytic cycling**

**program in end-stage kidney disease patients: a randomised controlled trial**

**Špela Bogataj¹^,^², Jernej Pajek¹^,^³, Jadranka Buturović Ponikvar¹^,^³, Vedran Hadžić², and Maja Pajek²^,*^**

¹University Medical Centre, Department of Nephrology, Ljubljana, 1000, Slovenia

²University of Ljubljana, Faculty of Sport, Ljubljana, 1000, Slovenia

³University of Ljubljana, Faculty of Medicine, Ljubljana, 1000, Slovenia

^*^[maja.pajek@fsp.uni-lj.si](mailto:maja.pajek@fsp.uni-lj.si)

SUPPLEMENTARY INFORMATION

Supplementary Table S1 Clinical parameters: blood pressure, body composition and biochemical results

| Variable | Group | At 8 weeks | At 16 weeks |
| --- | --- | --- | --- |
| Systolic blood pressure (mm Hg) | EXP | 141 ± 14 | 145 ± 18 |
|  | CON | 147 ± 19 | 146 ± 21 |
| Diastolic blood pressure (mm Hg) | EXP | 78 ± 11 | 79 ± 11 |
|  | CON | 85 ± 10 | 81 ± 10 |
| Lean tissue index (kg/m^2^) | EXP | 14.5 ± 2.5 | 14.5 ± 2.4 |
|  | CON | 13.3 ± 2.5 | 12.4 ± 1.9 |
| Fat tissue index (kg/m^2^) | EXP | 10.9 ± 4.1 | 10.9 ± 4.4 |
|  | CON | 11.9 ± 6.3 | 12.9 ± 6.1 |
| Serum pre-dialysis creatinine (qmol/L) | EXP | 788 ± 138 | 778 ± 141 |
|  | CON | 775 ± 181 | 749 ± 165 |

Note: Values are expressed as mean ± SD. Baseline values are given in Table 1. Blood pressure was defined as the mean of last three pre-dialysis blood pressure values. Abbreviations: EXP, experimental group; CON, control group.

**Supplementary Table S2** Exercises used in the functional training program

| **Warm-up** | **Main part** | **Cool-down** |
| --- | --- | --- |
| Light cardiovascular exercises and exercises for coordination and balance;  - walking in place  - walking heel to toe  - walking on toes  - walking backward  - knee lifts  - heel raises  - shoulder circles  - arm circles  - arm swings  - trunk twists  - leg swings forward  - ankle circles | - Squat: chair squat, wall squat with an exercise ball, squat, squat with dumbells  - Lunges: stationary lunge, lunge with a chair, walking lunge, reverse lunge, rear/front foot elevated lunge, lunge with dumbbells  - Push-ups: wall push-ups, incline push-ups, knee push-ups, regular push-ups  - Pull exercises: horizontal and vertical pull with a resistance band, trx, dumbbells  - Leg raises: back, and side raises (adjusting load with resistance bands and ankle weights)  - Pallof press, modified side plank, sit-ups, glute bridges, seated rotations, bird dog, chest squeeze with a medicine ball, dead bug | - palm press up toward the ceiling  - calf stretches  - abdominal stretch  - shoulder stretch  - hip flexor stretch  - lower back stretch  - quadriceps stretch  - biceps stretch  - seated forward bend  - knee-to-chest pose |
